# Image-guided percutaneous targeting of lymph nodes: a novel approach for salvage pelvic lymphadenectomy in recurrent prostate cancer

**DOI:** 10.1590/S1677-5538.IBJU.2017.0066

**Published:** 2017

**Authors:** Gustavo Caserta Lemos, Arie Carneiro, Guilherme Cayres Mariotti, Jose Roberto Colombo, Marcelo Apezzato, Marcelo Livorsi da Cunha, Fernado Cotait Maluf, Rodrigo Gobbo Garcia

**Affiliations:** 1 Hospital Israelita Albert Einstein - Oncology Center, São Paulo, Brasil

*To the editor,*

Recently, Torricelli et al. (1) published a video showing a step by step technique for salvage lymph node dissection after radical prostatectomy. With the development of novel imaging techniques, the identification of PCa patients with a clinical lymphonode (LN) relapse has become feasible. Salvage LN dissection (SLND) represents a treatment option for patients with prostate cancer relapse limited to the LN, with a potential beneficial impact of pelvic LN dissection on survival in these patients (2, 3). Usually a template extended SLND is performed, however the properly identification of the compromised LN is still a challenge and may be related to the treatment fail (3). We present a case of successful de novo SLND with image-guided percutaneous targeting LN using colloidal charcoal for recurrence detected by ^68^Ga-PSMA PET/CT following RP and previous salvage lymphadenectomy.

Our patient is a 52-year old man with PCa diagnosed by transrectal ultrasound guided biopsy (Gleason 4+3 in 2/14 cores and 3+3 in 3 cores) with PSA: 4.58ng/dL and negative CT and bone scan who underwent retropubic radical prostatectomy and limited LN dissection [Pathology: PCa Gleason 8 (4+4) and 7 negative LN]. One month post-operatively the urinary continence and erectile function were recovered with PSA: 0.18ng/dL and 0.22ng/dL after 3 months. ^68^Ga-PSMA PET/CT revealed positive LN in the right obturatory region. Open SLDN was performed displaying 7 free LN. One month post-operatively the PSA was still elevated (0.82ng/dL). A new ^68^Ga-PSMA PET/CT revealed the same suspected LN with higher SUV (Figure-1). De novo bilateral robotic SLDN was performed after percutaneous CT-guided targeting of ^68^Ga-PSMA PET/CT scan positive LN. The lesion was identified and 3mL of 4% solution of colloidal charcoal and lipiodol was injected into LN using a extraperitoneal lateral approach 20G needle (Figure-2). The rationale is to dilute a small amount of activated carbon into a thick substance to stabilize the material and prevent migration to adjacent structures, which may be an oil (such as ethiodinzed oil-lipiodol®) or a tissue adhesive (such as n-butyl-2-cyanoacrylate-histoacryl®) as we have preferred and recent data have been published (5). We found an inflammatory and stuck tissue around the blood vessels and ureter related with the two previous surgeries (Figure-3). In the right side an extended LN dissection was performed identifying the target LN previously tattoed close to the hypogastric artery distal to the umbilical artery. In the left side, a classic extended LN dissection was performed.

**Figure 1:**
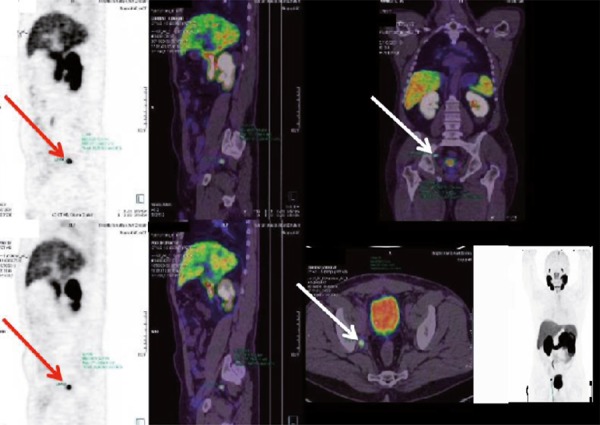
68Ga-PSMA PET/CT (fusion images) revealed an increased uptake in gallium Ga 68 (68Ga) - labeled PSMA (SUV=45.8) in suspected LN with 1.0cm in the right obturator fossa.

**FIGURE 2:**
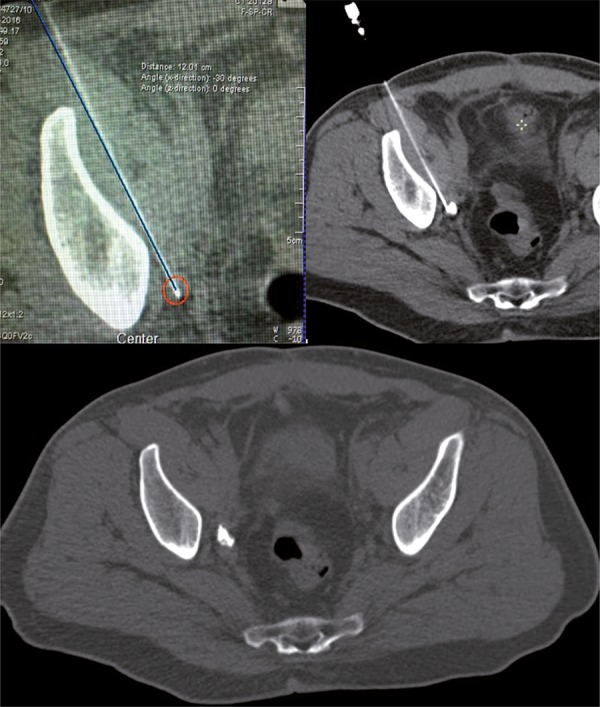
CT-GUIDED PERCUTANEOUS PUNCTURE OF THE LYMPH NODE USING A EXTRAPERITONEAL LATERAL APPROACH 20G NEEDLE AND 3ML OF SOLUTION OF 4% COLLOIDAL CHARCOAL AND LIPIODOL WAS INJECTED INTO THE LN.

**Figure 3:**
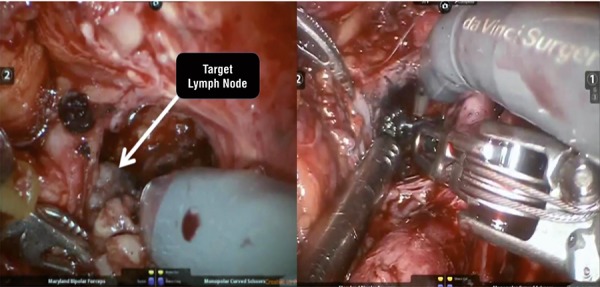
Target LN previously tattoed close to the hypogastric artery distal to the umbilical artery.

Our operative time was just under 3 hours with an estimated blood loss of 150mL. JP drain was maintained until discharge on postoperative day 2. There were no intraoperative or postoperative complications. The final pathology revealed 1 of 4 positive LN on the right side. Three months follow-up revealed PSA<0.04ng/dL.

Image-guided percutaneous targeting of ^68^Ga-PSMA PET scan positive LN is a safe, reasonable cost and useful technique in facilitating salvage pelvic lymphadenectomy for recurrence following radical prostatectomy, that may reduce cost by avoiding additional surgeries or radiotherapy. It helps to identify the target LN and may be related to the improvement of the outcomes in experienced hands.

## References

[1] Torricelli FC, Cividanes A, Guglielmetti GB, Coelho RF (2015). Robotic Salvage Lymph Node Dissection After Radical Prostatectomy. Int Braz J Urol.

[2] Tilki D, Mandel P, Seeliger F, Kretschmer A, Karl A, Ergün S (2015). Salvage lymph node dissection for nodal recurrence of prostate cancer after radical prostatectomy. J Urol.

[3] Abdollah F, Briganti A, Montorsi F, Stenzl A, Stief C, Tombal B (2015). Contemporary role of salvage lymphadenectomy in patients with recurrence following radical prostatectomy. Eur Urol.

[4] Abreu A, Fay C, Park D, Quinn D, Dorff T, Carpten J (2016). Robotic salvage retroperitoneal and pelvic lymph node dissection for ‘node-only’ recurrent prostate cancer: technique and initial series. BJU Int.

[5] Tyng CJ, Nogueira VH, Bitencourt AG, Santos LC, Souza TV, Zilio MB (2015). Computed tomographically guided injection of cyanoacrylate in association with preoperative radioguided occult lesion localization of ground-glass opacities. Ann Thorac Surg.

